# Probing SARS-CoV-2-positive plasma to identify potential factors correlating with mild COVID-19 in Ghana, West Africa

**DOI:** 10.1186/s12916-022-02571-2

**Published:** 2022-10-03

**Authors:** Kesego Tapela, Fatima O. Oyawoye, Charles Ochieng’ Olwal, Precious C. Opurum, Jones Amo Amponsah, Kekeli Aku Lumor Segbedzi, Becky Tetteh, Frederick Kumi-Ansah, Joe K. Mutungi, Evangeline Obodai, Emmanuella Amoako, Seth Agyemang, Nicaise Tuikue Ndam, William Kwabena Ampofo, Julian C. Rayner, Gordon A. Awandare, Lily Paemka, Yaw Bediako, Peter Kojo Quashie

**Affiliations:** 1 West African Centre for Cell Biology of Infectious Pathogens, College of Basic and Applied Sciences, University of Ghana, Legon, Accra, Ghana; 2 Department of Biochemistry, Cell and Molecular Biology, University of Ghana, Legon, Accra, Ghana; 3 Department of Immunology, Noguchi Memorial Institute for Medical Research, University of Ghana, Legon, Accra, Ghana; 4 Department of Microbiology, Cape Coast Teaching Hospital, Cape Coast, Ghana; 5 Virology Department, Noguchi Memorial Institute for Medical Research, University of Ghana, Legon, Accra, Ghana; 6 Department of Pediatrics, Cape Coast Teaching Hospital, Cape Coast, Ghana; 7 Yemaachi Biotech Inc., 222 Swaniker St, Accra, Ghana; 8 UMR261 MERIT and Head of IRD Branch in Benin-Nigeria-Togo-Ghana, Accra, Ghana; 9 Cambridge Institute for Medical Research, Cambridge Biomedical Campus, University of Cambridge, Cambridge, UK; 10 The Francis Crick Institute, 1 Midland Rd, London, NW1 1AT UK

**Keywords:** COVID-19, West Africa, Asymptomatic, ABO blood groups, Eotaxin, Antibodies

## Abstract

**Background:**

West Africa has recorded a relatively higher proportion of asymptomatic coronavirus disease 2019 (COVID-19) cases than the rest of the world, and West Africa-specific host factors could play a role in this discrepancy. Here, we assessed the association between COVID-19 severity among Ghanaians with their immune profiles and ABO blood groups.

**Methods:**

Plasma samples were obtained from Ghanaians PCR-confirmed severe acute respiratory syndrome coronavirus 2 (SARS-CoV-2)-positive individuals. The participants were categorized into symptomatic and asymptomatic cases. Cytokine profiling and antibody quantification were performed using Luminex™ multiplex assay whereas antigen-driven agglutination assay was used to assess the ABO blood groups. Immune profile levels between symptomatic and asymptomatic groups were compared using the two-tailed Mann-Whitney *U* test. Multiple comparisons of cytokine levels among and between days were tested using Kruskal-Wallis with Dunn’s post hoc test. Correlations within ABO blood grouping (O’s and non-O’s) and between cytokines were determined using Spearman correlations. Logistic regression analysis was performed to assess the association of various cytokines with asymptomatic phenotype.

**Results:**

There was a trend linking blood group O to reduced disease severity, but this association was not statistically significant. Generally, symptomatic patients displayed significantly (*p* < 0.05) higher cytokine levels compared to asymptomatic cases with exception of Eotaxin, which was positively associated with asymptomatic cases. There were also significant (*p* < 0.05) associations between other immune markers (IL-6, IL-8 and IL-1Ra) and disease severity. Cytokines’ clustering patterns differ between symptomatic and asymptomatic cases. We observed a steady decrease in the concentration of most cytokines over time, while anti-SARS-CoV-2 antibody levels were stable for at least a month, regardless of the COVID-19 status.

**Conclusions:**

The findings suggest that genetic background and pre-existing immune response patterns may in part shape the nature of the symptomatic response against COVID-19 in a West African population. This study offers clear directions to be explored further in larger studies.

**Supplementary Information:**

The online version contains supplementary material available at 10.1186/s12916-022-02571-2.

## Background

Coronavirus disease 2019 (COVID-19), caused by severe acute respiratory syndrome coronavirus 2 (SARS-CoV-2), is a global pandemic that has spread with unprecedented speed and is characterized by repeated waves driven by the emergence of new SARS-CoV-2 genetic variants [1]. COVID-19’s clinical spectrum ranges from asymptomatic, mild to severe illness [2]. COVID-19 in Africa has been unequal. COVID-19 fatalities remained low in Africa [3] till enhanced variants and enhanced vaccination elsewhere caused fatalities to rise in Africa. Even then Ghana and West Africa have consistently had lower case fatality rates compared to the rest of Africa even after considering variants of concern (Additional file 6). Southern Africa and parts of Eastern Africa have had high case counts and case fatality rates, while West African countries have largely had low fatalities, even in the face of high case counts [4]. Moreover, West Africa has also been affected by different SARS-CoV-2 variants [5, 6], like the rest of the world. Reports indicate that most West African countries have lower numbers of cases with severe outcomes and death, while at least 80% of cases appear to be asymptomatic [7–9]. Several factors, such as a lower average age and life expectancy [10], repeated exposure to other infections [11] and the warm climate [12], have been proposed to contribute to the apparent lower severity of SARS-CoV-2 in West Africa. Ghana and other West African countries such as Ivory Coast, Benin, Togo and Nigeria share similar climate/disease exposure and genealogy. Decreased diagnostic capacity has been reported to play a role in low reported COVID-19-related mortality. However, reduced testing would actually artificially inflate case fatality rates, since asymptomatic cases are mostly missed, whereas large-scale deaths are hard to miss [8, 13–15]. It is more likely that host factors may play a part in driving the different proportions of symptomatic and asymptomatic cases in West Africa relative to the rest of Africa and the world.

Severe COVID-19 illness manifests as acute respiratory distress syndrome, septic shock, multiple organ failure and intense cytokine production, a phenomenon termed cytokine storm [16]. The severity of COVID-19 may be influenced by age, comorbidities [17], host blood group [18] and underlying immune responsiveness, particularly the production of cytokines and antibodies [19–21]. COVID-19 severity has been in particular associated with cytokine storm, which leads to immune exhaustion, immune compromise and death [22]. A number of studies have shown that symptomatic patients have higher concentrations of cytokines compared to asymptomatic groups [23–26]. Specifically, inflammatory mediators such as interleukin (IL)-2, IL-6, IL-1, IL-7, IL-10, tumour necrosis factor (TNF), granulocyte colony-stimulating factor (G-CSF) and monocyte chemoattractant protein-1 (MCP-1) have been correlated with disease severity [25, 27–30]. Reports further show an increase of some cytokines, including TNF-α, interferon-gamma (IFN-γ) and IL-6, over time during infection [31]. Increased levels of immunoglobulin G (IgG) have also been observed, which plateaus after the 6th day among symptomatic patients [32, 33]. Moreover, significantly elevated levels of IgG against spike and nucleocapsid were found in symptomatic compared to asymptomatic cases [34, 35].

While there have been many studies of immune correlates of COVID-19 severity to date, there have not been any reported studies on the immune responses of West Africans. Considering the apparent differences in disease presentation between different geographic regions and the fact that several West African countries appear to have, at least so far, lower levels of severe COVID-19, a better understanding and characterization of the immune responses among African populations is urgently needed. This would help in the understanding of disease progression and could also provide insights into the use of cytokine inhibitors as potential treatments in different populations, and the use of cytokine biomarkers and/or seroprevalence testing for epidemiology in a West African context. This study focused on Ghana, a West African country with a relatively high incidence of SARS-CoV-2 infection and a high proportion of asymptomatic cases [8], similar to neighbouring countries.

We sought to investigate whether as reported elsewhere, if blood group, cytokine or SARS-CoV-2 antibody response is predictive of disease severity.

## Methods

### Study area, design and participants’ descriptions

Participants were recruited among the Ghanaian population during the ongoing COVID-19 pandemic. This study was a mixed methods study consisting of a baseline snapshot analysis (144) and longitudinal analysis for a subset of samples (61). The subsets were 58 randomly chosen individuals who were sampled up to 1 month and three individuals whose sampling extended to 4 months. These three individuals were a family, two of whom lived together. All participants were above the age of 4, were not pregnant and had received at least one positive COVID-19 test immediately prior to sample collection. Samples were obtained from the Ga East Regional Hospital in Accra and Cape Coast Teaching Hospital, in Cape Coast, Ghana. Additional samples used for blood group analysis were obtained from staff and students at West African Centre for Cell Biology of Infectious Pathogens, University of Ghana (WACCBIP-UG). Individuals with active SARS-CoV-2 infection were identified based on quantitative reverse transcription PCR (qRT-PCR) results, written informed consent obtained and blood samples collected. Blood samples were processed into plasma and peripheral blood mononuclear cells (PBMCs).

In total, 144 plasma samples were from COVID-19-positive individuals; of these, symptomatic (*n* = 29) and asymptomatic (*n* = 29) individuals were randomly chosen and sampled every week for 4 weeks to allow for characterization of immune responses over time. The major variants in circulation in Ghana, during the sampling period evolved from B.1 in 2020 to Delta, in July 2021 [5]. At the date the last participant was recruited (11 July 2021), there were only 92,562 confirmed COVID-19 cases in Ghana (https://www.worldometers.info/coronavirus/country/ghana/). Thus, we present here, data related to 0.3% of all confirmed cases, as at time of sampling. This makes our study modestly representative of the Ghanaian outbreak of SARS-CoV-2, despite the apparently small sample size.

Symptomatic participants were defined as those who tested positive for SARS-CoV-2 with COVID-19 symptoms while asymptomatic cases were defined as individuals who tested positive for SARS-CoV-2 but had no reported COVID-19 symptoms. The patient’s disease status was taken from medical reports and through a questionnaire. Control plasma used for ABO blood grouping (*n* = 267) was obtained from healthy (non-COVID-19 positive) consented individuals recruited at WACCBIP-UG. Negative infection controls were from pre-COVID plasma samples collected between 2017 and 2018 (*n* = 100) from blood donors at Korle Bu teaching hospital (Accra, Ghana) blood bank and from individuals who tested negative for SARS-CoV-2 infection during the ongoing pandemic (2020–2021, *n* = 33).

### Sample collection and preparation

Blood (5 ml) was collected into ethylenediamine tetraacetic acid (EDTA) tubes using the venepuncture method. Baseline samples were collected immediately after PCR confirmation of SARS-CoV-2 infection, or concurrently with sample acquisition for PCR, then included into the study once a positive result had been obtained. Plasma was isolated from whole blood using centrifugation as previously described with minor modifications [35]. Briefly, whole blood samples were centrifuged for 5 min at 1000g. Plasma from the interface was collected and stored at −80°C for subsequent analyses. The cellular components of the blood were further processed and stored for future use.

### ABO blood group typing

Antigen-driven agglutination on a microtitre plate format was used to identify participants’ ABO blood groups [36]. First, antigen reagents were prepared using plasma from healthy controls. The blood antigen type (antigens A and B) for the control samples was determined using an anti-sera kit (Maxwin Healthcare Pvt Ltd, Chennai, India) following the manufacturer’s protocol. Control plasma designated as A or B were pooled and stored at 4°C as a 3% (v/v) red cell concentration in normal saline solution. To determine the blood group of participants, 100 μl of participant plasma was aliquoted into microplate wells followed by the addition of 50 μl of 3% antigen A reagent. The same procedure for the same plasma ID was repeated in a different well with antigen B. Once the plate was full, it was centrifuged at 1000g for 1 min at 25°C and the pellet resuspended using a micropipette. Agglutination was determined macroscopically and implied the presence of antibodies against the reagent antigen used [36]. The presence of agglutination when antigen A was used indicated anti-A antibodies; hence, the sample could be B or O. The same criteria were applied for antigen B. Samples showing agglutination when both antigens were used were designated as blood group O while samples with no agglutination regardless of antigen were grouped as group AB.

### Immune activation profiling

Cytokines and chemokine levels in participant plasma were used as an indicator of immune activation and response [37]. Plasma samples were processed using a Cytokine Human Magnetic 25-plex panel assay (Invitrogen, Thermo Fisher Scientific). This panel included cytokines which have been found to be associated with COVID-19 severity elsewhere [25, 27–29] and also represents the major categories of cytokines. The kit monitored cytokines: IL-1Ra, IL-1β, IL-2, IL-2R, IL-4, IL-5, IL-6, IL-8, IL-10, IL-10, IL-12, IL-13, IL-15 and IL-17A, IFN-α, IFN-β, colony-stimulating factor (GM-CSF) and TNF-α, and chemokine family (RANTES, Eotaxin, MIP-1α, MIP-1β, MIG, MCP-1 and IP-10). Briefly, 100-μl plasma samples and antibody beads were added to a 96-well plate and incubated for 2 h. The plates were then washed 3 times while the beads were being mobilized using a magnetic holder. Biotinylated antibody was added and incubated for 1 h followed by 30-min incubation with streptavidin-Rhodophyta Phycoerythrin. The plates were read using a Luminex MAGPIX system (Luminex Corporation, Austin, TX, USA) using xPONENT™ software (V.4.3.229) following the manufacturer’s protocol.

### SARS-CoV-2-specific antibody quantification

The levels of antibodies against SARS-CoV-2 nucleocapsid and spike proteins were estimated as previously described [38]. Beads coupled to SARS-COV-2 nucleocapsid and spike, following a protocol(hal-01299922 [39] (courtesy of National Research Institute for Sustainable Development, France, https://en.ird.fr/), were used in this study. To eliminate cross-reactive responses, nucleocapsid and spike antigen from SARS-CoV-1 and MERS-CoV were also included. The coupled beads (25 μl of each bead) were distributed into a 96-well plate. The supernatant was removed while beads were mobilized using a magnetic holder and blood plasma (1:100) was added to the plate. The plate was incubated for 2 h at 25°C on a plate shaker (400 rpm), then washed. An anti-human lgG secondary antibody (4μg/ml) was added to the plate and incubated for 30 min in a Corning® LSE™ digital microplate shaker (400 rpm). This was followed by 3 washes, addition of streptavidin-phycoerythrin (1μg/ml) (Invitrogen) and incubation for 10 min in a plate shaker (400 rpm). Finally, the plates were read using a Luminex MAGPIX system (Luminex Corporation, Austin, TX, USA) using xPONENT™ software (V.4.3.229).

### Data and statistical analysis

Raw data was saved in Excel format and data analysis was performed using GraphPad prism software Inc version 9 (GraphPad Software, San Diego, CA, USA), Stata 16 (StataCorp, College Station, TX, USA) and open resource packages anchored on R software version 4.1.0 (R Development Core Team, Vienna, Austria, and R studio Version 2022.2.0.443). The Gaussian distribution of data was assessed by the Shapiro-Wilk normality test. Since data were not normally distributed, immune profile levels between symptomatic and asymptomatic groups were compared using the two-tailed Mann-Whitney *U* test. Multiple comparisons of cytokines levels between days were tested using Kruskal-Wallis with Dunn’s post hoc test and presented as median with (25th and 75th percentiles). The plasma half-life of cytokines in asymptomatic and symptomatic patients was analysed using a decay curve with time (days) on the *X*-axis and cytokine magnitude was on the *Y*-axis. The relationship between ABO blood grouping (O’s and non-O’s) and cytokines was determined using the Pearson chi-square test. Correlations between cytokines were determined using Spearman correlations. Univariate analysis was performed to assess the association of various cytokines with asymptomatic phenotype. For univariate and multivariate analyses, cytokine levels were classified as high (represented by 1) and low (represented by 0). The median cytokine levels of samples from healthy individuals were used as cut-offs to identify high or low levels (Table S1). A multivariable logistic model was obtained by using a backward stepwise procedure. Variables that were associated with asymptomatic at the *p* < 0.25 level were included. A *p* value < 0.05 was considered statistically significant.

## Results

### Participant’s characteristics and sample usage

**Fig. 1:**
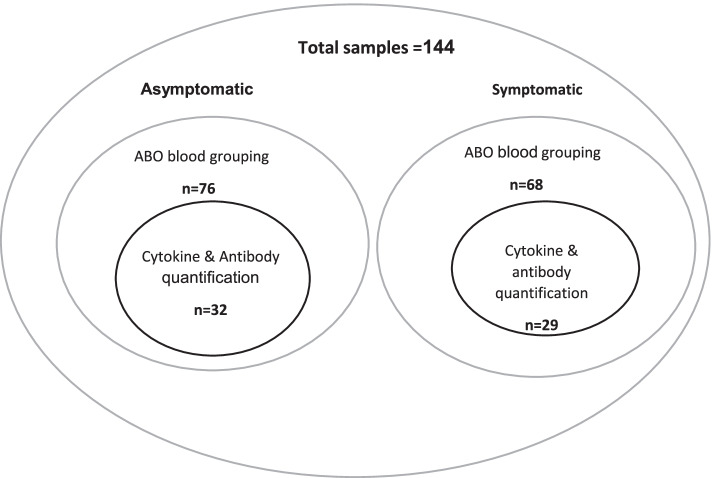
Sample distribution for different assays performed in the study

A hundred and forty-four participants were recruited for all arms of this study, excluding the controls. Based on available metadata, participants were classified as asymptomatic (76), symptomatic (66) and non-survivors (2). ABO blood group typing was performed on baseline samples from all 144 individuals. Longitudinal samples, up to 1 month post-diagnosis for 58 individuals and 4 months post-diagnosis for 3 individuals were available for profiling of cytokine and SARS-CoV-2 antibody levels. The three individuals were a family and two of the participants lived together (Fig. 1; Table 1). Control plasma used for ABO blood grouping (*n* = 267) was obtained from healthy consented individuals recruited at the West African Centre for Cell Biology of Infectious Pathogens, University of Ghana (WACCBIP-UG), while plasma samples (*n* = 100) collected between 2017 and 2018 from healthy blood donors at Korle Bu teaching hospital blood bank were used as a control for cytokine profiling.

**Table 1:** The characteristics of study participants

	Baseline (*n* = 144)	Longitudinal samples (*n* = 61)
Median age, years (IQR)^a^	35 (13–78)	35 (21–76)
Male	71 (49%)	29 (48%)
Female	73 (51%)	32 (52%)
SpO_2_^b^	98 (94.5–98.75)	
Clinical status		
Asymptomatic	76	32
Symptomatic	66	29
Non-survivors	2	0

^a^When data was available

^b^Symptomatic cases only

### A trend exists between ABO blood group O and asymptomatic COVID-19

**Fig. 2:**
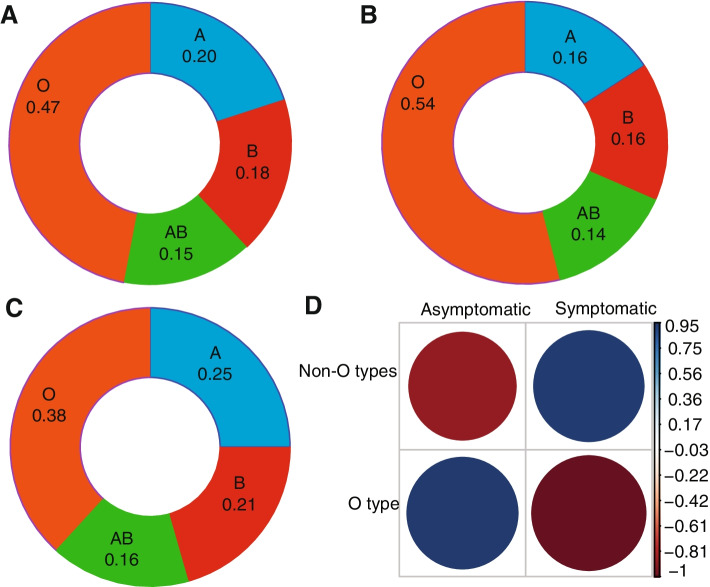
The distribution of blood group within asymptomatic and symptomatic and correlations between blood group and disease status. The distribution of the blood group is presented as ratios in a donut plot for all samples (**A**), asymptomatic (**B**) and symptomatic (**C**) and the correlations between blood group and disease status were presented as correlograms (**D**). The magnitude of Pearson residuals is shown on a colour scale of dark blue (positive correlation, 0.95–0.03) to white (no correlation) to red (negative correlation, −0.15 to −0.88) signal. The bigger the “dot”, the stronger or weaker the correlation coefficient

To investigate the relationship between blood group distribution and the occurrence of asymptomatic and symptomatic COVID-19 in our study population, the reverse blood typing method was utilized [36]. The blood group distribution in the study population in ascending order was AB (15.3%), B (18.1%), A (20.1%) and O (46.5%). Blood group O was thus the most common blood group (Fig. 2A) as expected, given its frequency in West Africa [40]. Compared to the general sample population, the proportion of individuals with blood group O was higher in asymptomatic (54%) compared to symptomatic cases (38%). Similarly, non-blood group O was disproportionately higher in the symptomatic group (Fig. 2B, C). There was a trend towards high rates of symptomatic infection in non-blood group O and higher rates of asymptomatic infection in blood group O (Fig. 2D). However, despite these trends, there was no statistically significant association between blood group and disease severity (*χ*^2^ = 2.958, *p* = 0.09).

### Asymptomatic SARS-CoV-2 individuals have lower levels of multiple cytokines

**Fig. 3:**
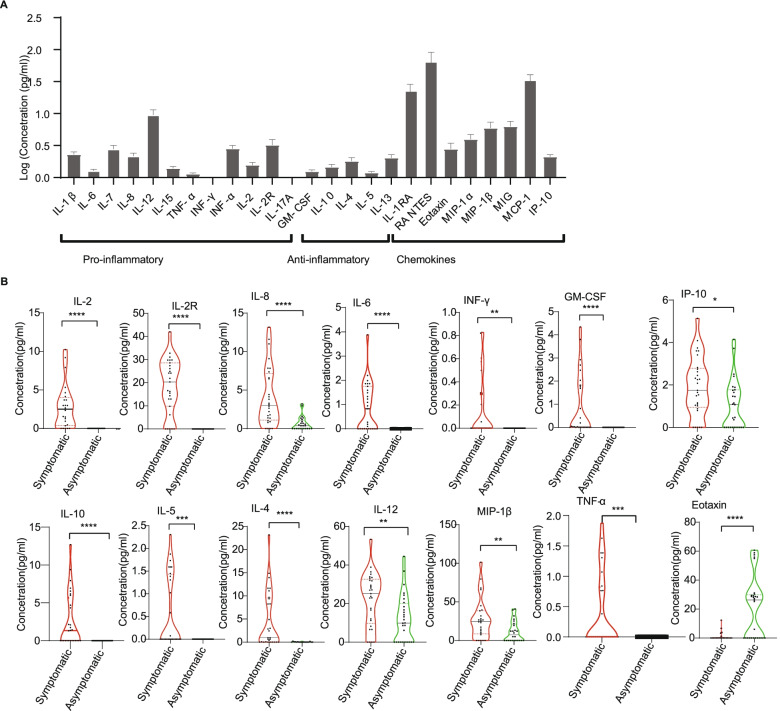
Cytokine concentration levels in asymptomatic and symptomatic cases. **A** Cytokine abundance among all COVID-19 patients and **B** comparison of cytokine concentration levels between COVID-19 symptomatic and asymptomatic patients. The cytokine concentration levels were measured from plasma of COVID-19 (*n* = 61) patients which were collected weekly for 4 weeks (medians of the time points). The bars depict the mean ± standard error of mean (SEM). The cytokine concentration levels were measured from plasma of COVID-19 symptomatic (*n* = 29) and asymptomatic (*n* =32) individuals. The median cytokine quantities per participant category obtained by extracting the medians across the time points per patient are shown by a horizontal line across the violin plots while the lower and upper dotted lines represent the 25th and 75th percentiles, respectively. Statistical significance between symptomatic and asymptomatic patients were determined by a two-tailed Mann-Whitney *U* test (ns: *p* >0.05, *p <0.05, **p < 0.01, ****p < 0.0001

To explore associations between cytokine abundance and infection outcome, we quantified cytokines in the plasma of SARS-COV-2-positive individuals (*n* = 61, roughly evenly split between asymptomatic and symptomatic, see Fig. 1). All participants displayed <10 pg/ml of most pro-inflammatory cytokines assessed, including IFN-γ, IL-6 and TNF-α, and anti-inflammatory cytokines such as IL-10, IL-4 and IL-5 (Fig. 3A; Additional file 4). These findings generally suggested a low inflammatory immune response among Ghanaian SARS-COV-2-infected individuals. There were however differences in plasma cytokine concentration levels between symptomatic COVID-19-positive patients and asymptomatic cases. Symptomatic COVID-19 patients had significantly (*p* < 0.05) higher levels of IL-10, IL-2, IL-2R, IL-6, IL-8 GM-CSF IP-10, TNF-α, IL-4, IL-5, IL-12, IFN-γ and MIP-1β compared to the asymptomatic cases (Fig. 3B). Higher levels of IFN-α, IL-1Ra, MIG, RANTES and IL-7 were also observed in symptomatic patients compared to asymptomatic cases, but these differences were not statistically significant (*p* > 0.05; Additional file 1; Additional file 4). There was also no difference in the levels of IL-13 (*p* = 0.47) and IL-1β (*p* = 0.98; Additional file 1) between the symptomatic and asymptomatic patients. Interestingly, only Eotaxin was significantly higher in asymptomatic compared to symptomatic cases (*p* = 0.001; Fig. 3B).

### Plasma cytokines persist in symptomatic individuals longer than asymptomatic participants

**Fig. 4:**
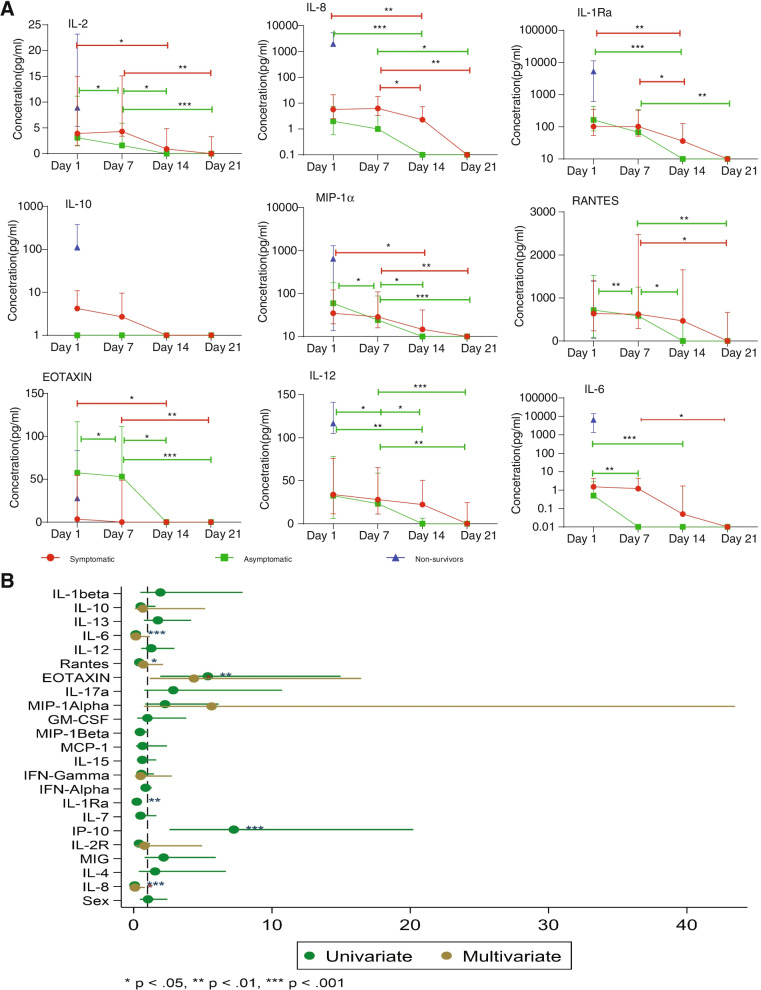
Change in cytokine concentration levels over time and cytokine association in COVID-19-positive individuals. **A** The cytokine concentration levels were analysed from plasma of COVID-19 symptomatic (*n* = 29), asymptomatic (*n* = 29) and non-survivors (*n* = 2) patients at different timepoints for 4 weeks. The median quantity of the cytokines for each sampling day is shown by the line graph and the 25th and 75th percentiles are cytokine levels. Statistical significance between days was determined by Kruskal-Wallis (*p <0.05, **p < 0.01, ****p < 0.0001). **B** The association of the cytokines and antibodies to asymptomatic phenotype were estimated using odds ratios with 95% confidence intervals were computed and presented

To assess the kinetics of cytokine expression, the level of the cytokines was evaluated weekly for 1 month among SARS-CoV-2-positive individuals. Additionally, 3 individuals were randomly chosen and followed up for 4 months to assess the kinetics more than a month (Additional file 3). The levels of IL-12, RANTES, MIP-1β, MCP-1, IL-1Ra, IL-2, IP-10, IL-2R, MIG, IL-4 and IL-8 decreased significantly over time for both asymptomatic and symptomatic individuals. However asymptomatic cases returned to baseline levels faster than symptomatic cases (Fig. 4, Additional file 5). For instance, while the levels of IL-2 steadily declined in both sets of patients over time, IL-12 levels in asymptomatic cases returned to baseline by day 14, while symptomatic patients returned to baseline by day 21. The same trend was seen for IL-6, IL-1Ra and RANTES (Fig. 4A). Interestingly, IL-10 appeared to be elevated in symptomatic patients only, returning to baseline by day 14. Eotaxin was the only cytokine among those measured that showed elevated levels in asymptomatic cases relative to symptomatic patients; these levels also decreased over time. Individuals who died due to COVID-19 complications (non-survivors) were characterized by elevated levels of most cytokines relative to the other patients (Fig. 4A). Individuals sampled for 4 months also showed a decreased cytokine expression over a month. For example, cytokines like IL-2, IL-12 and MIP-1β for samples A and B decreased over a month, remained constant for at least a month and returned to baseline (Additional file 2). In general, cytokine levels in asymptomatic cases return to baseline levels faster than in symptomatic individuals.

### Eotaxin was strongly associated with asymptomatic cases

To determine the association between cytokines and asymptomatic phenotype, logistic regression analyses were performed on all the samples. Samples were divided into high and low by comparison with median levels of cytokines levels in healthy pre-COVID individuals (Table S1). Asymptomatic individuals were significantly (*p* < 0.05) more likely to have higher levels of Eotaxin (odds ratio, OR: 4.4, 95% CI: 1.2–16) and low levels of IL-6 (OR: 0.16, 95% CI: 0.058–0.42), IL-8 (OR: 0. 0.074, 95% CI: 0.02–0.8) and IL-1Ra (OR: 0.023, 95% CI: 0.08–0.8) (Fig. 4B). Taken together, the present findings imply that Eotaxin, IL-8, I-1Ra and IL-6 are associated with the asymptomatic phenotype.

### Cytokine clustering patterns differ between symptomatic and asymptomatic cases

**Fig. 5:**
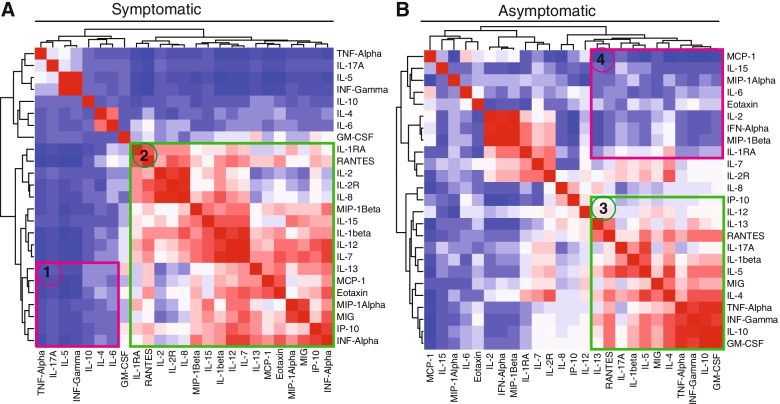
Cytokine correlations in individuals with COVID-19. Clustering between symptomatic and asymptomatic COVID-19-positive individuals. The cytokine clustering patterns are shown in heatmaps for both symptomatic and asymptomatic patients. The magnitude of correlation coefficients is shown on a colour scale of red (strong positive correlation), white (no correlation) to dark blue (strong negative correlation) signal. The difference in clustering patterns between symptomatic and asymptomatic is shown in selections 1, 2, 3 and 4

Given the apparent association between cytokine dynamics and clinical presentation in SARS-CoV-2-infected patients in our study population, we sought to establish whether specific combinations of cytokine levels could be used to distinguish between COVID-19 symptomatic and asymptomatic individuals. Therefore, correlations between individual cytokines within each group were calculated and presented as heatmaps (Fig. 5A).

This analysis revealed that the asymptomatic group shared a diffuse network that was a combination of cytokines IFN-α, IL-7 and IL-12 with chemokines MCP-1, Eotaxin, MIG and MIP-1α. These were characterized by weak negative and positive correlations between the cytokines (cluster 1). Another network of inflammatory cytokines including TNF-α, IFN-γ, GM-CSF, IL-4 and IL-5 with strong negative correlations was observed among asymptomatic cases (cluster 2).

In the symptomatic group, a cluster of positively correlated cytokines (TNF, IFN-γ, GMCSF and IL-10), which is typically associated with inflammation (cluster 3) was observed. Moreover, a group of highly negatively correlated cytokines (IL-15, IL-2 and IFN-α) was also shown (cluster 4). The heatmap and the clustering dendrogram confirmed the presence of specific cytokine patterns depending on the severity of the disease status (symptomatic or asymptomatic).

### Antibody expression levels between symptomatic and asymptomatic cases

**Fig. 6:**
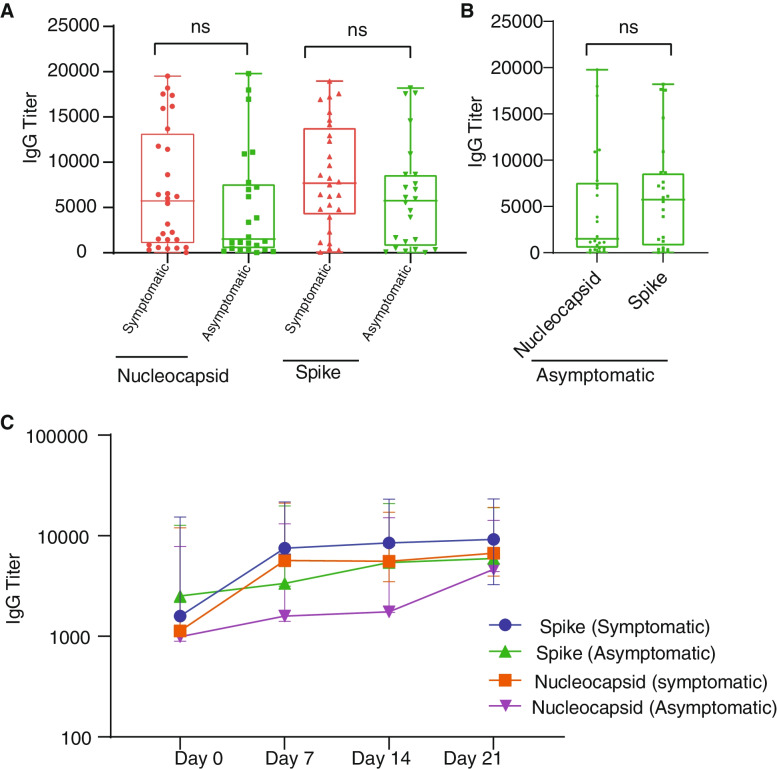
Antibody profiles among SARS-CoV-2-infected patients. Differential expression levels of IgG against SARS-CoV-2 spike and nucleocapsid proteins in symptomatic and asymptomatic persons (**A**) and differential expression levels of IgG against SARS-CoV-2 spike and nucleocapsid proteins in asymptomatic cases (**B**). The kinetics of IgG in symptomatic and asymptomatic individuals (**C**) in response to SARS-CoV-2. Data represents the median quantity of antibody quantities per participant category obtained by extracting the medians across the time points per patient and 25th and 75th percentiles

To compare antibody levels between symptomatic and asymptomatic patients, antibody titres against SARS-CoV-2 spike and nucleocapsid were quantified at baseline (*t* = 0) for all samples, and a subset of individuals were followed for 1 month and 4 months to assess the antibody level kinetics over time. Median antibody levels were higher in symptomatic patients compared to asymptomatic cases, but this difference was not statistically significant (Fig. 6A). Plasma levels of IgG in symptomatic patients increased from day 1 then plateaued after day 7 for both proteins while in asymptomatic cases the rate of increase over time was more modest (Fig. 6C). When three related individuals who live together were followed for 4 months, antibody declined over a month but spiked for all three after 60 days—likely a case of re-infection (Additional file 3). Levels of anti-spike antibodies were generally higher than anti-nucleocapsid antibodies in both symptomatic and asymptomatic individuals, albeit not significant (*p* = 0.34; *p* = 0.50, Fig. 6B; Additional file 3).

## Discussion

To date, reported mortality and case numbers of COVID-19 infection in West Africa have remained markedly lower compared to other regions. However, the underlying factors responsible for the differential mortalities among West Africans remain unclear, with many hypotheses put forward. Considering the centrality of host immunity in COVID-19 prognosis [40] and the importance of blood groups in viral infections [41], the present study sought to probe plasma samples from SARS-CoV-2-positive Ghanaians to identify correlates of COVID-19 severity among West Africans.

In our study, there was no significant association between the ABO blood group and COVID-19 severity. However, there was a trend towards higher rates of asymptomatic infection in blood group O and high rates of symptomatic infection in non-blood group O, which is in line with previous studies in Turkey [42], China [41] and the USA [43], all which found trends but no significant correlations of blood type with disease severity and deaths. Since blood group O prevalence ranges between 47 and 50% [44, 45] in Ghana, it is possible that the high cases of mild or asymptomatic COVID-19 individuals in Ghana may be in part attributable to the dominance of blood group O in the population. It has been reported that SARS-CoV-2 receptor-binding domain (RBD) potentially prefers binding to antigen A, expressed on respiratory epithelial cells, than O [46]. This may cause inflammation responses in blood group A individuals but not in blood group O; thus, this may be protective of blood O individuals.

We observed generally low levels (≤10pg/ml) of pro-inflammatory and anti-inflammatory cytokines among SARS-COV-2-positive individuals. These are cytokines that have been reported to be associated with the severity and pathogenesis of COVID-19 [24, 47, 48]. A concentration of more than 37 pg/ml of IL-6 was reported to be predictive of COVID-19 severity and death [49], which is far higher than the concentration level of IL-6 found by our study. Importantly, cytokine levels in asymptomatics were frequently lower than in healthy pre-COVID-19 samples. Our data suggests that COVID-19-positive Ghanaian populations may have lower COVID-19-induced cytokine levels compared to other populations. This implies that the lower mortalities experienced in Ghana may in part, be due to decreased incidence of cytokine storm, which is a major cause of severity and deaths among COVID-19 patients in other locations [50–52]. While the baseline level of cytokines was lower in our overall study population, in agreement with previous findings [23, 24], we did observe higher levels of multiple cytokines in symptomatic patients than asymptomatic cases, as well as lower levels of IL-1Ra, IL-6 and IL-8 associated with the asymptomatic phenotype. Since these are mostly Th1 cytokines, it is possible that asymptomatic individuals have reduced Th1 inflammation immune responses, resulting in reduced symptoms [53]. West Africans frequently suffer from malaria and other infectious diseases. It may be that chronic exposure to malaria and other pathogens may have induced strong regulatory immune responses that counteract excessive non-specific inflammation [10, 54].

Furthermore, we found that plasma levels of most of these cytokines declined over time in both symptomatic and asymptomatic patients. Our small cohort of 3 individuals who were followed for 4 months also showed a decrease over time. The increases observed after 60 days were likely due to re-infection since a concomitant increase in antibody levels was observed. One key finding is that cytokine responses declined steadily during our period of monitoring, compared to studies done elsewhere, where cytokine levels increased steadily [31, 54, 55]. Our non-survivors, however, had higher levels of cytokines which seemed consistent with cytokine storm [31, 54, 55], though we only had a single time-point sample for them. One caveat is we could not be sure of the time of infection for most of our samples, and as such, we were not sure of the stage of the disease. Given the time-sensitive nature of obtaining a positive SARS-CoV-2 test [56, 57], we were moderately confident that our baseline samples reflected early infection in most cases. However, the fact that even our symptomatic cohort showed declines in cytokines over time implied that this population is responding better and may not easily experience severe cytokine-related COVID-19 complications. There is no evidence of cytokine storm, maybe even cytokine dampening, similar to what is observed with the use of steroidal drugs [58]. Considering our modest sample size, more in-depth studies with a larger cohort are warranted to confirm these findings.

Notably, Eotaxin was the only cytokine with significantly higher concentration levels in asymptomatic than symptomatic patients. Findings from the USA also detected higher concentrations of this chemokine in COVID-19 survivors than non-survivors [59, 60]. Furthermore, the study detected an association of Eotaxin with asymptomatic phenotype. Eotaxin is a potent eosinophil chemoattractant within the bronchial wall of asthmatic patients. Eosinophils help in the destruction of pathogens using granules [61]. It is possible that higher expression of this chemokine among asymptomatic cases in our study population deters progression to severe disease.

The study further explored the correlations of these cytokines and showed that cytokines relate differently between symptomatic and asymptomatic patients. Unique cytokine correlation clusters were identified in both symptomatic and asymptomatic individuals. Inflammatory cytokines such as IL-6, IL-15 and IL-8 were highly correlated with one another in symptomatic cases while their expression appeared less coordinated in asymptomatic cases. Since the West African population is mostly dominated by asymptomatic cases, we suggest lower cytokine levels in the population may be due to dysregulated or attenuated cytokine responses. These correlations might be causing decreased levels of cytokines in asymptomatic patients.

With respect to antibody profiles in the context of COVID-19, we observed an initial increase in antibody production levels, which plateaued after day 7 in our study population, followed by a gradual decline over 30 days. The plateauing trend is in line with previous reports from China [32] and France [62], where patients were followed for 19 and 22 days respectively. However, the antibodies waned after a month. Other studies have also shown a decrease in antibody levels after a month [63, 64].

## Conclusions

This report provides new insights on the SARS-CoV-2 immune response in the West African population, which can be explored further in larger studies. This study showed evidence that a combination of host biological factors is linked to mild presentations of COVID-19 in Ghana: (i) a dampened cytokine response and (ii) a strong antibody response at baseline to SARS-CoV-2 spike and nucleocapsid protein. Further study will be focused on cell populations associated with symptomatic and asymptomatic infections, the impact of different variants/waves and single-cell sequencing to fully decipher the impact of potential modulators of the immune response such as *Plasmodium* sp. infection.

The main limitation of the study was the sample size (*n* = 144). However, these findings hypothesize about the role of cytokines regulation in reducing COVID-19 severity in West Africa and call for further mechanistic investigations to clearly establish the key mediators which could be exploited for immunotherapeutic interventions.

Another limitation is that we were limited to peripheral blood, not blood from organs such as the lungs. As such, we cannot rule out that cytokine and antibody responses may differ at virus-host interfaces in the lungs and other organs. However, peripheral blood is a good reflector of the state of the whole body, these results are still a good indicator of the status of immune function against COVID-19 disease in the West African context.

## Supplementary Information

**Additional file 1. **Cytokine concentration levels in COVID-19 patients. A: Comparison of cytokine concentration levels between COVID-19 symptomatic and asymptomatic patients. The median quantity of the cytokines is shown by a horizontal line across the violin plots while the lower and upper dotted lines represent the 25^th^ and 75^th^ percentiles, respectively. B: Change on cytokine concentration levels over time in COVID-19 positive individuals. The cytokine concentration levels were measured from plasma of COVID-19 symptomatic (*n* = 29) and asymptomatic (*n* = 29) individuals. Statistical significance between symptomatic and asymptomatic patients were determined by a two-tailed Mann-Whitney U test. (*: *p* <0.05, **: *p* < 0.01, ****: *p* < 0.0001, ns: *p* >0.05).

**Additional file 2. **Change on cytokine concentration levels over time in COVID-19 positive individuals. The cytokine concentration levels analysed from plasma of COVID-19 asymptomatic (*n* = 3), monthly for four months. The quantity of the cytokines for each sampling month is shown by the line graph.

**Additional file 3. **Antibody profiles among SARS-CoV-2 infected patients. A: The kinetics of IgG in asymptomatic individuals (*n* = 3) in response to SARS-CoV-2. Data represents the quantity of the multiple time points of the cytokines, B: Differential expression levels of IgG against SARS-CoV-2 spike and nucleocapsid proteins in symptomatic in response to COVID-19. Data represents the median quantity with the 25^th^ and 75^th^ percentiles.

**Additional file 4. **Cytokine concentration levels in COVID-19 patients. Comparison of cytokine concentration levels between COVID-19 symptomatic, asymptomatic patients, pre-COVID-19 health participants, COVID-19 pandemic health individuals and COVID-19 non-survivors. The cytokine concentration levels were measured from plasma of COVID-19 symptomatic (*n* = 29) and asymptomatic (*n* = 29), individuals, pre-COVID-19 health participants (100), COVID-19 pandemic health individuals (33) and COVID-19 non-survivors (2). The median quantity of the cytokines is shown by a horizontal line across the scatter plot while the lower and upper dotted lines represent the 25^th^ and 75^th^ percentiles, respectively. Statistical significance between symptomatic and asymptomatic patients were determined by a Kruskal-Wallis test with Dunn’s post hoc. (*: *p* <0.05, **: *p* < 0.01, ****: *p* < 0.0001, ns: *p* >0.05).

**Additional file 5. **The half-life of cytokines in asymptomatic and symptomatic patients. The cytokine concentration levels were for cytokines with significant difference between symptomatic and asymptomatic cases (14 cytokines), pro-inflammatory, anti-inflammatory and chemokines. The median half-life of the cytokines is shown by a horizontal line across the dot plot while the lower and upper dotted lines represent the 25^th^ and 75^th^ percentiles, respectively. Statistical significance between symptomatic and asymptomatic patients were determined by Mann-Whitney test (*: *p* <0.05, **: *p* < 0.01, ****: *p* < 0.0001, ns: *p* >0.05).

**Additional file 6.** Coronavirus disease 2019’s fatality rate in the world and Africa. The data was retrieved from WHO COVID-19 dashboard (https://covid19.who.int/). The COVID-19 vaccine was rolled out in December 2020 worldwide and in February/March 2021 in Africa.

**Additional file 7. **The Pearson correlation coefficient (r) between concentration levels of cytokines with significant difference between symptomatic and asymptomatic cases (14 cytokines), and baseline viral loads (Ct value) of the patients at *p* value<0.05.

**Additional file 8: Table S1.** The median cytokine levels of healthy individuals (*n* = 124). **Table S2.** Heathy control samples used in the study.

## Data Availability

The datasets used and/or analysed during the current study are available from the corresponding author on reasonable request.

## Abbreviations

EDTA: Ethylenediamine tetraacetic acid
GM-CSF: Granulocyte macrophage colony-stimulating factor
IFN-α: Interferon-alpha
IFN-β: Interferon-beta
IgG: Immunoglobulin G
IL: Interleukin
IP-10: Induced protein of 10 kDa
MCP-1: Monocyte chemoattractant protein-1
MIG: Monokine induced by interferon-gamma
MIP-1α: Macrophage inflammatory protein-1α
MIP-1β: Macrophage inflammatory protein-1β
PBMC: Peripheral blood mononuclear cells
qRT-PCR: Quantitative reverse transcription PCR
Th1: T-helper type 1
TNF-α: Tumour necrosis factor
WACCBIP-UG: West African Centre for Cell Biology of Infectious pathogens, University of Ghana
