# Supplementary figures and images for: Probing SARS-CoV-2-positive plasma to identify potential factors correlating with mild COVID-19 in Ghana, West Africa
Source: BMC Med. 2022 Oct 3;20:370. doi: 10.1186/s12916-022-02571-2 (PMC9527094; doi:10.1186/s12916-022-02571-2)

A

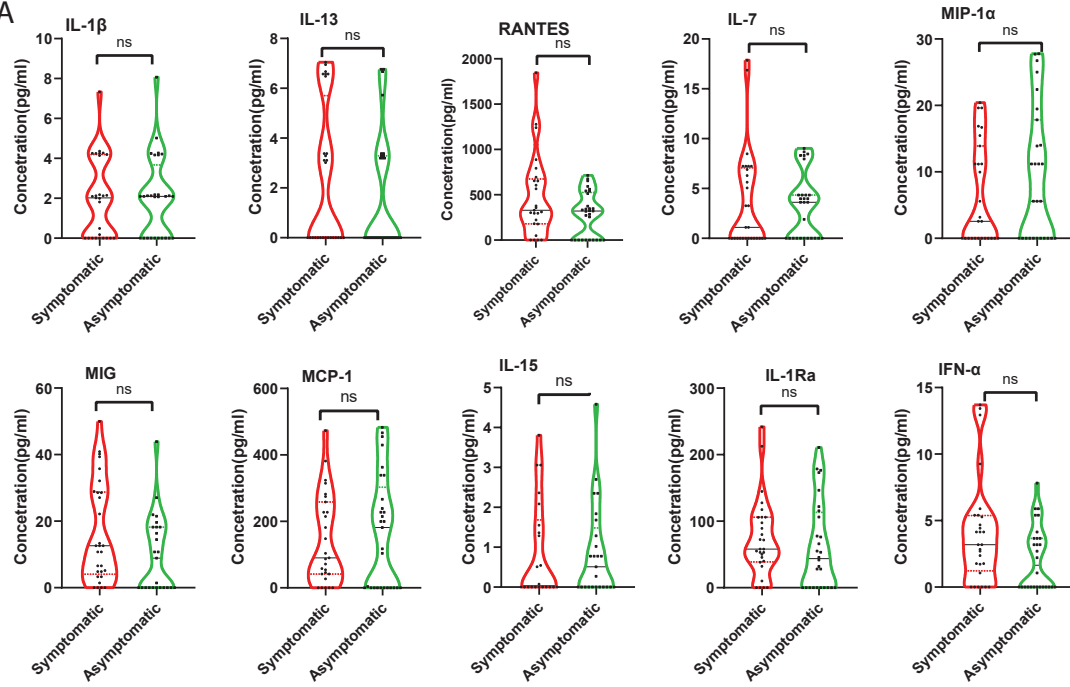

B

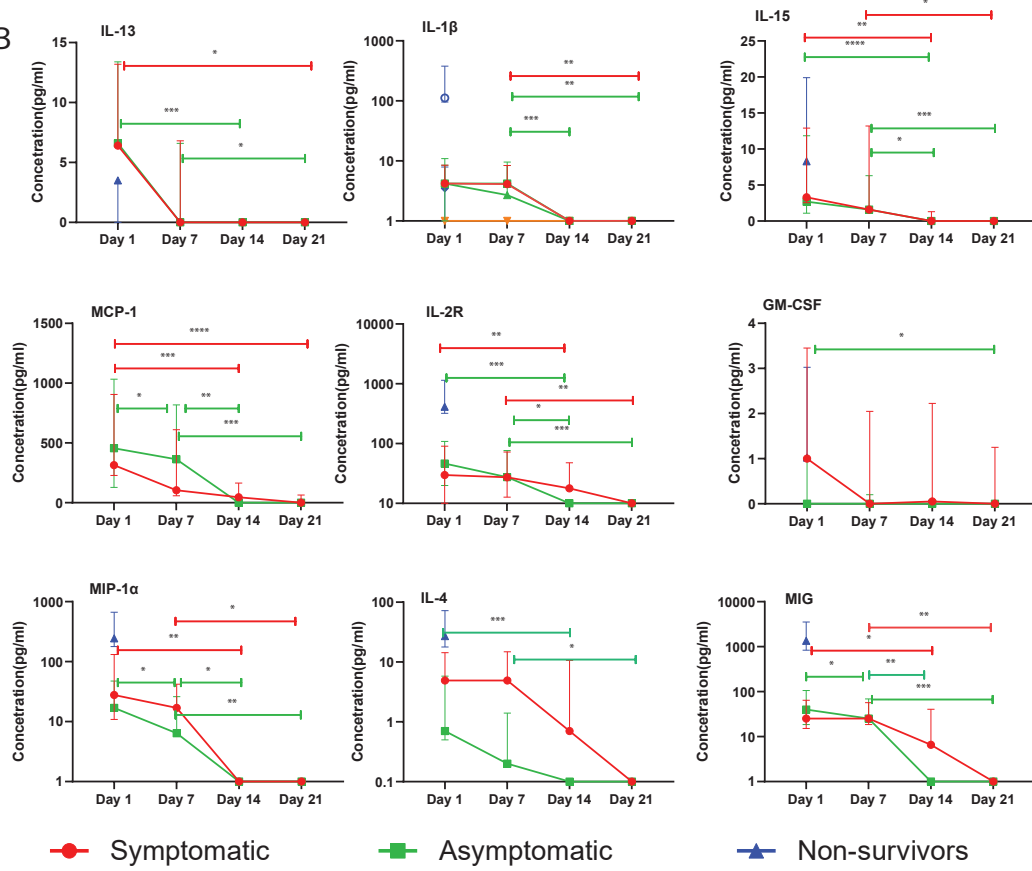

● Symptomatic

■ Asymptomatic

▲ Non-survivors

Supplement: Supplementary file 1 — Additional file 1. Cytokine concentration levels in COVID-19 patients. A: Comparison of cytokine concentration levels between COVID-19 symptomatic and asymptomatic patients. The median quantity of the cytokines is shown by a horizontal line across the violin plots while the lower and upper dotted lines represent the 25th and 75th percentiles, respectively. B: Change on cytokine concentration levels over time in COVID-19 positive individuals. The cytokine concentration levels were measured from plasma of COVID-19 symptomatic (n = 29) and asymptomatic (n = 29) individuals. Statistical significance between symptomatic and asymptomatic patients were determined by a two-tailed Mann-Whitney U test. (*: p <0.05, **: p < 0.01, ****: p < 0.0001, ns: p >0.05). [file 12916_2022_2571_MOESM1_ESM.pdf]

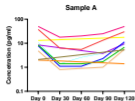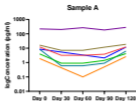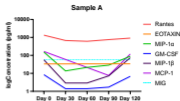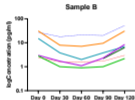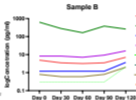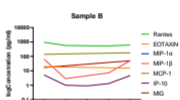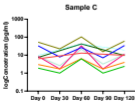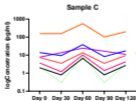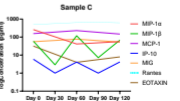

Supplement: Supplementary file 2 — Additional file 2. Change on cytokine concentration levels over time in COVID-19 positive individuals. The cytokine concentration levels analysed from plasma of COVID-19 asymptomatic (n = 3), monthly for four months. The quantity of the cytokines for each sampling month is shown by the line graph. [file 12916_2022_2571_MOESM2_ESM.pdf]

A

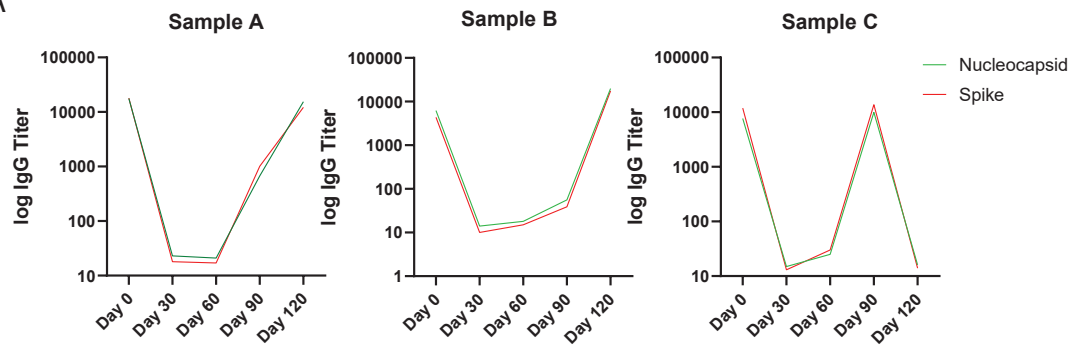

B

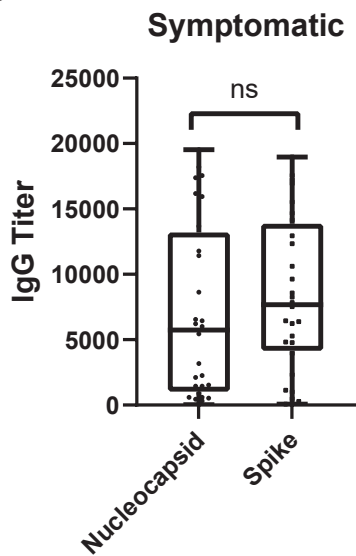

Supplement: Supplementary file 3 — Additional file 3. Antibody profiles among SARS-CoV-2 infected patients. A: The kinetics of IgG in asymptomatic individuals (n = 3) in response to SARS-CoV-2. Data represents the quantity of the multiple time points of the cytokines, B: Differential expression levels of IgG against SARS-CoV-2 spike and nucleocapsid proteins in symptomatic in response to COVID-19. Data represents the median quantity with the 25th and 75th percentiles. [file 12916_2022_2571_MOESM3_ESM.pdf]

**A**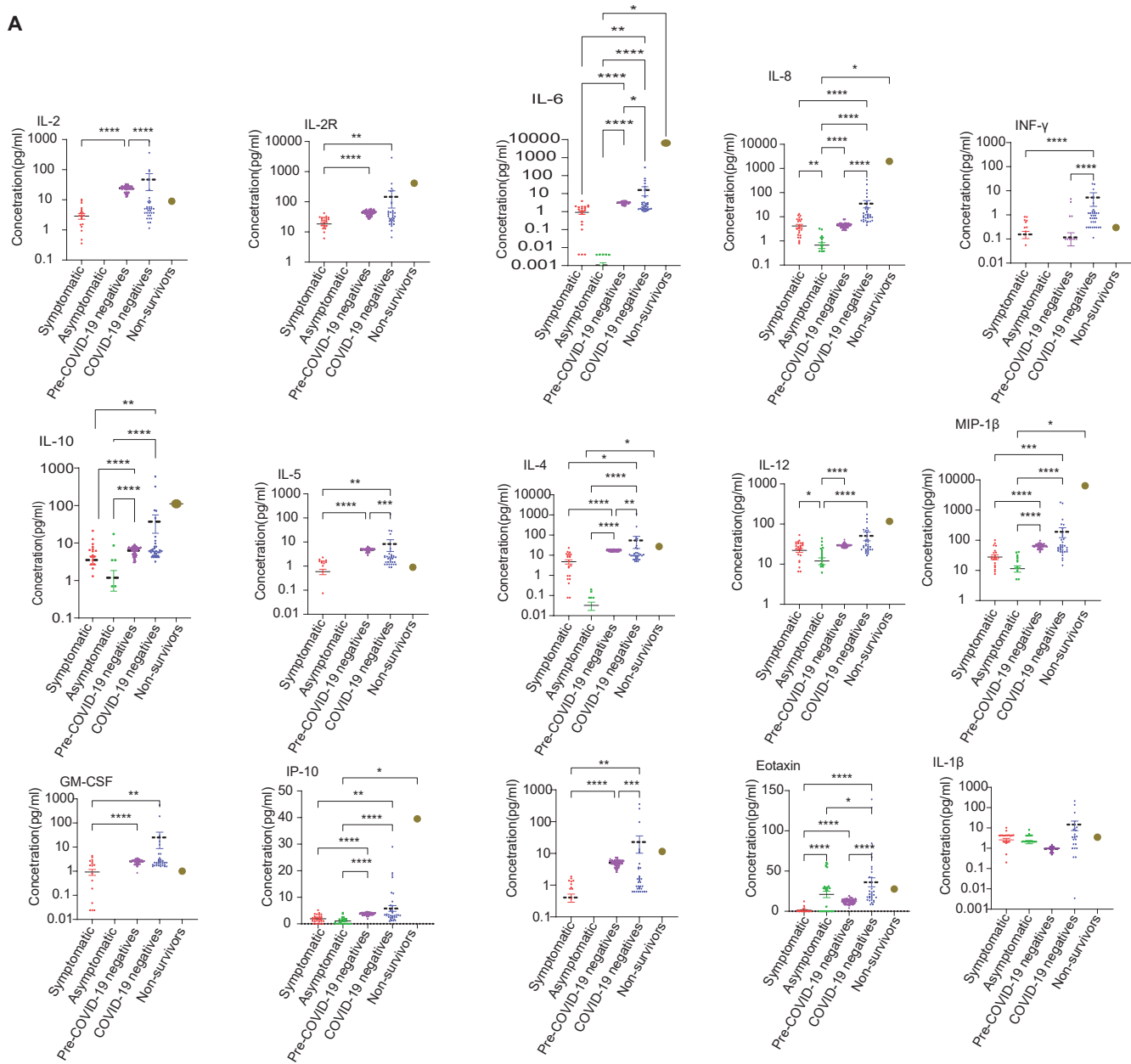**B**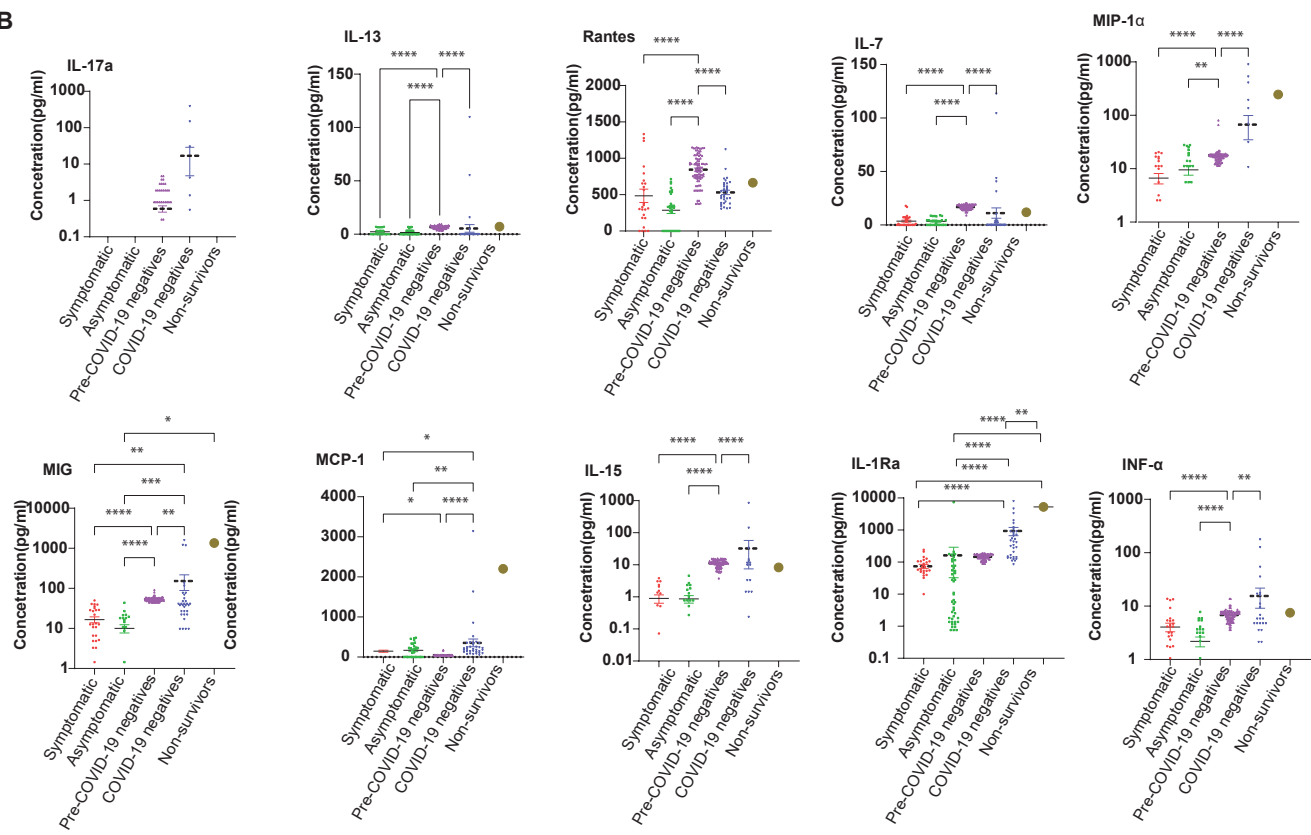

Supplement: Supplementary file 4 — Additional file 4. Cytokine concentration levels in COVID-19 patients. Comparison of cytokine concentration levels between COVID-19 symptomatic, asymptomatic patients, pre-COVID-19 health participants, COVID-19 pandemic health individuals and COVID-19 non-survivors. The cytokine concentration levels were measured from plasma of COVID-19 symptomatic (n = 29) and asymptomatic (n = 29), individuals, pre-COVID-19 health participants (100), COVID-19 pandemic health individuals (33) and COVID-19 non-survivors (2). The median quantity of the cytokines is shown by a horizontal line across the scatter plot while the lower and upper dotted lines represent the 25th and 75th percentiles, respectively. Statistical significance between symptomatic and asymptomatic patients were determined by a Kruskal-Wallis test with Dunn’s post hoc. (*: p <0.05, **: p < 0.01, ****: p < 0.0001, ns: p >0.05). [file 12916_2022_2571_MOESM4_ESM.pdf]

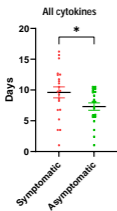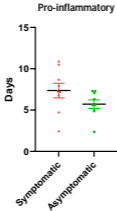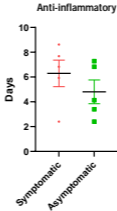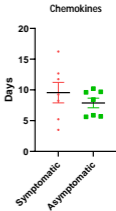

Supplement: Supplementary file 5 — Additional file 5. The half-life of cytokines in asymptomatic and symptomatic patients. The cytokine concentration levels were for cytokines with significant difference between symptomatic and asymptomatic cases (14 cytokines), pro-inflammatory, anti-inflammatory and chemokines. The median half-life of the cytokines is shown by a horizontal line across the dot plot while the lower and upper dotted lines represent the 25th and 75th percentiles, respectively. Statistical significance between symptomatic and asymptomatic patients were determined by Mann-Whitney test (*: p <0.05, **: p < 0.01, ****: p < 0.0001, ns: p >0.05). [file 12916_2022_2571_MOESM5_ESM.pdf]

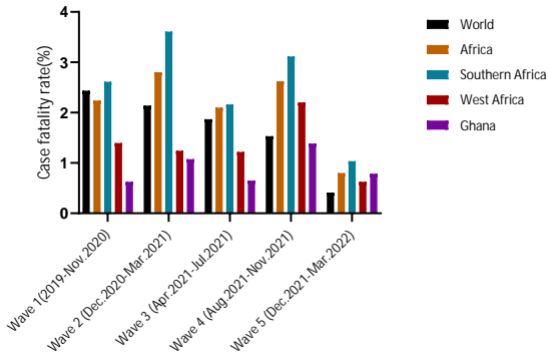

Supplement: Supplementary file 6 — Additional file 6. Coronavirus disease 2019’s fatality rate in the world and Africa. The data was retrieved from WHO COVID-19 dashboard (https://covid19.who.int/). The COVID-19 vaccine was rolled out in December 2020 worldwide and in February/March 2021 in Africa. [file 12916_2022_2571_MOESM6_ESM.pdf]

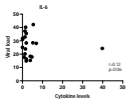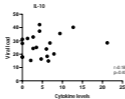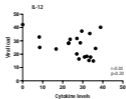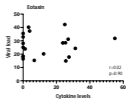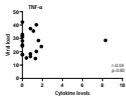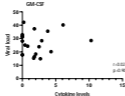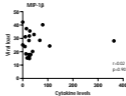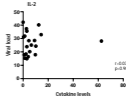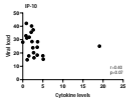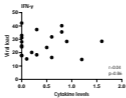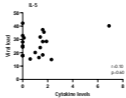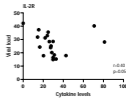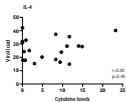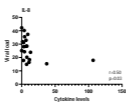

Supplement: Supplementary file 7 — Additional file 7. The Pearson correlation coefficient (r) between concentration levels of cytokines with significant difference between symptomatic and asymptomatic cases (14 cytokines), and baseline viral loads (Ct value) of the patients at p value<0.05. [file 12916_2022_2571_MOESM7_ESM.pdf]
