# Supplementary material for: Probing SARS-CoV-2-positive plasma to identify potential factors correlating with mild COVID-19 in Ghana, West Africa
Source: BMC Med. 2022 Oct 3;20:370. doi: 10.1186/s12916-022-02571-2 (PMC9527094; doi:10.1186/s12916-022-02571-2)
Supplement: Supplementary file 8 — Additional file 8: Table S1. The median cytokine levels of healthy individuals (n = 124). Table S2. Heathy control samples used in the study. [file 12916_2022_2571_MOESM8_ESM.docx]

**Supplementary tables**

**Table S1**. **The median cytokine levels of healthy individuals(n=124)**

| **Cytokine/Chemokine** | **Median levels** |
| --- | --- |
| IL-1β | 1 |
| IL-10 | 6 |
| IL-13 | 6 |
| IL-6 | 3 |
| IL-12 | 29 |
| RANTES | 762 |
| Eotaxin | 13 |
| IL-17a | 1 |
| MIP-1α | 17 |
| GM-CSF | 3 |
| MIP-1β | 64 |
| MCP-1 | 47 |
| IL-15 | 11 |
| IL-5 | 5 |
| IFN-γ | 1 |
| IFN-α | 7 |
| IL-1Ra | 159 |
| TNF-α | 5 |
| IL-2 | 24 |
| IL-7 | 16 |
| IP-10 | 4 |
| IL-2R | 43 |
| MIG | 51 |
| IL-4 | 18 |
| IL-8 | 5 |

**Table S2: Heathy control samples used in the study**

|  | **ABO blood grouping** | **Pre-COVID negatives** | **COVID-19 negatives** |
| --- | --- | --- | --- |
| **N** | 267 | 100 | 33 |
| **Age range** | 18-78 | 18-45 | 18-78 |
